# Comparing genome scans among species of the stickleback order reveals three different patterns of genetic diversity

**DOI:** 10.1002/ece3.8502

**Published:** 2022-01-24

**Authors:** James Reeve, Qiushi Li, Dorothea Lindtke, Samuel Yeaman

**Affiliations:** ^1^ Department of Biological Sciences University of Calgary Calgary Alberta Canada; ^2^ Present address: Tjärnö Marina Laboratorium Göteborgs Universitet Strömstad Sweden; ^3^ Present address: Institute of Chinese Materia Medica China Academy of Chinese Medical Sciences Beijing China; ^4^ Present address: Institute of Plant Sciences University of Bern Bern Switzerland

**Keywords:** comparative genomics, convergent evolution, genome scan, stickleback

## Abstract

Comparing genome scans among species is a powerful approach for investigating the patterns left by evolutionary processes. In particular, this offers a way to detect candidate genes that drive convergent evolution. We compared genome scan results to investigate if patterns of genetic diversity and divergence are shared among divergent species within the stickleback order (Gasterosteiformes): the threespine stickleback (*Gasterosteus aculeatus*), ninespine stickleback (*Pungitius pungitus*), and tubesnout (*Aulorhynchus flavidus*). Populations were sampled from the southern and northern edges of each species’ range, to identify patterns associated with latitudinal changes in genetic diversity. Weak correlations in genetic diversity (*F*_ST_ and expected heterozygosity) and three different patterns in the genomic landscape were found among these species. Additionally, no candidate genes for convergent evolution were detected. This is a counterexample to the growing number of studies that have shown overlapping genetic patterns, demonstrating that genome scan comparisons can be noisy due to the effects of several interacting evolutionary forces.

## Graphical abstract

Genome scans are popular tools for detecting shared evolutionary events among species. However, not all scans will show overlapping results. This article detects three different outcomes of genome scans, and few shared genetic patterns.

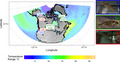

## 1 INTRODUCTION

Genome scans are useful tools for identifying the effects of evolutionary processes on the genome of a species (Fraser & Whiting, 2020; Lotterhos & Whitlock, 2015). In the past decade they have been used to analyze genomic patterns in many wild species (Alves et al., 2019; Dennenmoser et al., 2017; Jones et al., 2012; Vijay et al., 2016; Westram et al., 2014), as they can provide genetic information about evolution without requiring typically impractical experimental setups. The growth of studies using genome scans has provided a new opportunity to compare results among species to identify common patterns of genetic variation, which may be imprinted on different species through the same evolutionary processes. Ultimately, comparisons of genome scans among species will help to assess the generality of genetic patterns to learn how evolution shapes the genomes of different species.

At the simplest level, genome scans are a comparison of genetic diversity among different populations within a species. Genetic diversity can be split into two main types; diversity within a population and diversity among populations (referred to as genetic divergence). Many statistics represent genetic diversity (e.g., π, *H*_E_, Tajima’s *D*, and Fay & Wu’s H) or genetic divergence (e.g., *F*_ST_, d_xy_), and different interpretations of these scores have been discussed at length in other papers (Burri et al., 2015; Ellegren et al., 2012; Reid et al., 2016; Van Doren et al., 2017; Vijay et al., 2016, 2017). A genome scan moves along the genome looking for extreme patterns of these statistics that may be associated with local adaptation (Fraser & Whiting, 2020; Lotterhos & Whitlock, 2015), but alternatively could be the product of background selection (Charlesworth et al., 1993; Matthey‐Doret & Whitlock, 2019) or demographic events such as range expansions, population bottlenecks, or inbreeding (Barton, 1998; Excoffier & Ray, 2008; Lotterhos & Whitlock, 2014; Nielsen et al., 2007). These extreme patterns can be identified visually as “peaks” and “troughs” of genetic diversity or divergence, from their distinctive shape on a Manhattan plot. Statistical methods are used to determine which evolutionary processes most likely generated these peaks and troughs, often as the first step towards identifying candidate genes.

Comparison of genome scan results among species provides insight into how shared ancestry, demography, and environmental conditions can affect the similarity of patterns in their genomes. Commonly, genome scans are compared to detect convergent evolution (Fraser & Whiting, 2020), as shared peaks or troughs have the potential to reveal genes that underpin evolution to a shared environmental pressure in many species (Stern, 2013). Examples of these convergently evolving genes have already been found such as digestive proteins in primates (Stewart et al., 1987), pigmentation in vertebrates (Gompel & Prud’homme, 2009; Hoekstra, 2006; Manceau et al., 2010) or anthocyanin proteins in flowering plants (Kopp, 2009). Outside of convergent evolution, comparing genome scans can also show shared properties of the genome such as recombination landscapes (Samuk et al., 2017) or ancestral population structure (Vijay et al., 2017). On one hand, genomes scans should not be used in isolation to detect convergent evolution, as shared patterns can come from several sources. On the other hand, genome scans offer a useful way to identify broad scale genetic similarities among several species. By comparing patterns in diversity and divergence across many species and environmental gradients, we can better understand how evolutionary processes affect the genome.

Threespine stickleback (*Gasterosteus aculateus*) is a good system for comparative genome scans, as several regions of the genome have been identified that are strongly associated with local adaptation in this species (Colosimo et al., 2005; Hohenlohe et al., 2010; Jones et al., 2012; Schluter & Conte, 2009). Several closely related fish species live in overlapping niches allowing their genomic landscape to be compared to the threespine stickleback’s to learn how evolution shapes patterns in their respective genomes. This study aims to compare patterns of genetic diversity and divergence in the threespine stickleback with both the ninespine stickleback (*Pungitus*; for simplicity, the stickleback species will be referred to as threespines and ninespines) and tubesnout (*Aulorhychus flavidus*), as an example of how comparisons of genome scan results can identify common genetic patterns.

Ninespines and threespines diverged 26 mya (Varadharajan et al., 2019) and have already been subjected to comparative genetic studies (Nelson & Cresko, 2018; Shapiro et al., 2009; Shikano et al., 2013; Varadharajan et al., 2019), in part because both species have colonized freshwater lakes in similar regions. Interestingly, while targeted genetic studies support convergent evolution to freshwater (Shikano et al., 2013), whole genome data found no genetic signatures of convergent evolution (Raeymaekers et al., 2017). The extent of similarity in genetic patterns among these sticklebacks is still an open question.

We are only beginning to compare the genomes of the threespine and tubesnout (in review) and have yet to explore the patterns of genetic diversity. These species diverged approximately 50 mya (Betancur et al., 2013), which is a timeframe similar to a study in birds which found similar patterns of genetic diversity maintained across 55 million years (Vijay et al., 2017). In contrast to the ninespine–threespine comparison, tubesnouts are an exclusively marine species that overlaps with the marine threespine along most of its range in the Pacific. Marine threespines are known to have genetic structure along the North American West coast (Morris et al., 2018), which may be the result of gene flow from locally adapted freshwater populations (Nelson & Cresko, 2018). Thus, we may expect to find patterns in the threespine genome that differ from the tubesnout’s, due to differences in their demographic history, selection, and ancestral variation.

Here, we compare patterns of population genomic diversity and divergence in these species to assess how such patterns vary across the stickleback order. Specifically, we study patterns in *F*_ST_ and genetic diversity from populations at each end of a latitudinal gradient and compare these patterns among species‐pairs at a whole‐genome and a gene‐by‐gene level to assess their similarity and test for signatures of convergent evolution. We focus on latitude‐related effects (e.g., adaptation in traits related to body size, growth rate, changing breeding times, or oxygen binding [Andersen et al., 2009; Bell & Foster, 1994, pp. 155–157; Blanck & Lamouroux, 2006]) instead of the patterns of salinity‐driven adaptation more commonly investigated in threespine and ninespine, as the tubesnout has not evolved to live in freshwater systems. By studying broad‐scale patterns that covary with the selection pressures associated with latitude, we aim to detect whether patterns of genetic diversity are shared among these species, to learn how evolution may have shaped such patterns.

## 2 METHOD

### 2.1 Sampling

**FIGURE 1:**
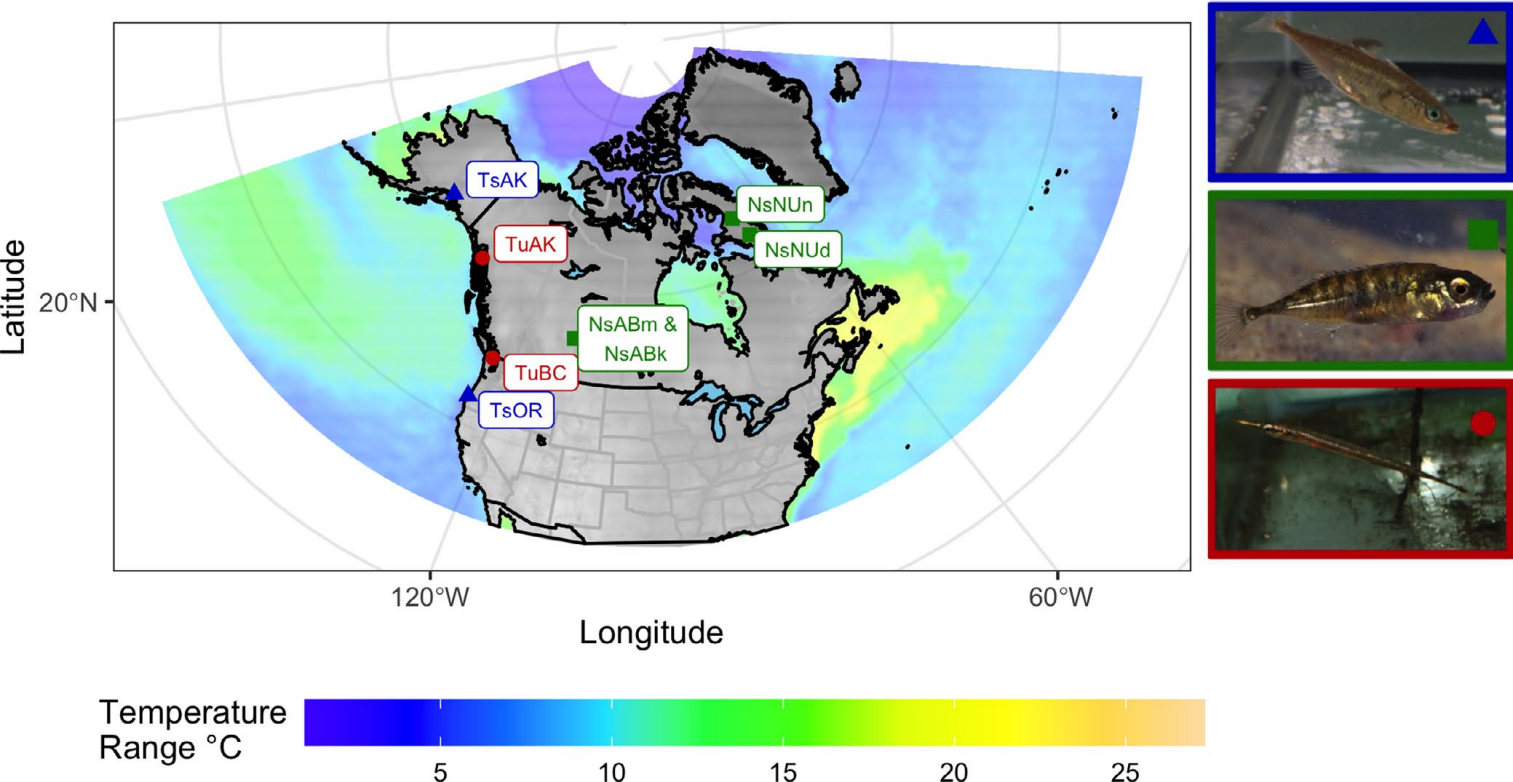
The sampling location for each species of fish. Threespine locations are represented by green triangles, ninespines by blue squares and tubesnouts by orange circles. Labels for each population are used consistently throughout this paper; the first half of the label denotes the species (Ts = threespine stickleback, Tu = tubesnout and Ns = ninespine stickleback). The second half of the label denotes the state or province where the population was collected (AK = Alaska, USA; BC = British Columbia, Canada; OR = Oregon, USA; AB = Alberta, Canada; NU = Nunavut, Canada). The two Albertan ninespine populations are combined into a single point (NsABm & NsABk) for visual clarity. The base map is projected in Azimutahl equal distances (datum = WGS84) orientated to center on Canada (latitude = 90 & longitude = −98.4). Ocean water is colored by the annual range in sea surface temperature (°C) taken from the Bio‐ORACLE database (Assis et al., 2018; Tyberghein et al., 2012). The final plot was compiled in R using the sf, ggplot, raster and grid packages

Tubesnout and threespine samples were collected between May and August 2017 from the West Coast of North America using dip netting and minnow traps. These fish were euthanized in the field using a mixture of 0.5 g/L MS222 (Ethyl 3‐aminobenzoate methanesulfonate) in sea water, the carcases were then preserved in 95% ethanol which was replaced after 24 h. The northern populations of both species and all ninespine samples were donated by collectors. Between 30 and 52 fish were collected per population (Table 1), the specific details of sampling locations are included in Table S1, and population labels are described in Figure 1.

**TABLE 1:** Average genetic diversity, standard error (*SE*) and population size (*N*) per population

Population	*N*	Number of windows	${\bar{H}}_{E}$	*SE*	Number of SNPs	*F*_ST_	*SE*
Threespine stickleback							
TsAK	52	8764	2.65 × 10^−3^	1.52 × 10^−5^	3,928,772	–	
TsOR	51		3.41 × 10^−3^	1.75 × 10^−5^			
Species average		–	3.03 × 10^−3^	1.43 × 10^−5^	–	0.14	9.98 × 10^−4^
*Tubesnout*							
TuAK	44	8925	2.93 × 10^−3^	1.73 × 10^−5^	3,466,658	–	
TuBC	50		2.86 × 10^−3^	1.21 × 10^−5^			
Species average		–	2.90 × 10^−3^	1.18 × 10^−5^	–	0.12	4.92 × 10^−4^
*Ninespine stickleback*							
NsNUn	46	15,058	0.41 × 10^−3^	0.67 × 10^−5^	687,627	*F*_ST_ only based on north‐south population pairs	
NsNUd	42		0.37 × 10^−3^	0.83 × 10^−5^			
NsABk	30		0.18 × 10^−3^	0.44 × 10^−5^			
NsABm	41		0.13 × 10^−3^	0.40 × 10^−5^			
Species average		–	0.27 × 10^−3^	0.57 × 10^−5^	–	0.49	1.41 × 10^−3^

Note

Genetic diversity is represented by both the average *F*_ST_ per SNP and average ${\bar{H}}_{E}$ per window for each population and each species. The population labels are explained in the caption of Figure 1.

### 2.2 DNA extractions and sequencing

DNA was extracted from a ~2‐mm clip of the pectoral fin of each fish using a Qiagen DNeasy Blood and Tissue Kit. The protocol was modified slightly to increase yield by washing the fins in dH_2_O before lysis and by repeating the elution step twice using half the volume of buffer. The DNA samples were checked for fragmentation using gel electrophoresis, quality tested with an Implen N60 Nanophotometer, and concentration was measured using a Qubit 3.0 with three replicates per sample. Samples with low quality (A260/A280 < 1.8; A260/A230 < 2.0) or low quantity (concentration <8 ng/μl) were re‐extracted. Any sample that failed three re‐extractions was removed. This quality check was repeated after pooling DNA samples (see below).

Individual DNA samples were pooled together by population before library preparation (see Table S2 for quality scores of pools). The DNA pools were sent to Genome Québec (McGill University and Génome Québec Innovation Centre, Montréal, Canada) for library preparation and 150bp paired‐end whole genome shotgun sequencing on their Illumina HiSeqX platform. The estimated coverage of each pool was set as double the number of individuals in the sample (2Nx), so that ideally each chromosome of each individual was sequenced once. A PCR step was performed with 318 cycles, even though it is not advised for Pool‐seq protocols (Schlötterer et al., 2014), because the mass of DNA in the pools did not meet Genome Québec’s minimum threshold for PCR‐free sequencing.

### 2.3 Bioinformatics

Unless otherwise mentioned, the default parameter settings were used for all software. Sequenced reads were trimmed of adaptors with Trimmomatic (v0.38; Bolger et al., 2014), using the paired‐end mode “PE” and with a minimum length set to 120bp. Further trimming was deemed unnecessary after inspecting read quality with FastQC (v0.11.7; Andrews, 2018)). Trimmed reads were mapped onto each species genome (threespine: Peichel et al., 2017; tubesnout: Q. Li & S. Yeaman, unpublished data; ninespine: Nelson & Cresko, 2018) with BWA‐ MEM (v0.7.12; Li & Durbin, 2009). PCR‐duplicates were flagged using Picard‐MarkDuplicates (v2.18.7; Broad Institute, 2018). As a prerequisite before running MarkDuplicates, the reads were sorted and read group information was added with Picard—AddOrReplaceReadGroups. Reads were realigned around indels to adjust quality scores for sites surrounding indels using GATK3—IndelRealigner (v3.8‐1‐0; McKenna et al., 2010). Before indel realignment the ninespine reads files, which were sequenced on separate lanes, were combined into a single file per population using samtools – merge (v1.9; Li, 2011; Li et al., 2009). After indel realignment samtools—mpileup was used to combine reads from all populations within a species. Any reads flagged as duplicates were ignored by samtools. VarScan (v2.3.9; Koboldt et al., 2012) was used to call SNPs for each species. The ploidy for each sample was set as double the number of individuals in the pool (2N). Thresholds were set to filter out multiallelic SNPs, low coverage (cov < 50), quality (qual < 20), minor alternative allele frequency (maf < 0.01), and SNPs with less than two reads for the minor allele (min‐read‐count <2). The coverage filter was set to ensure that each individual in a sample was represented at least once, assuming DNA pooling was balanced.

### 2.4 Genetic diversity calculations

Genetic diversity was measured as two different values, the genetic diversity within a population (${\bar{H}}_{E}$) and the genetic divergence (*F*_ST_). *F*_ST_ was calculated for each species using the R package *poolFstat* (Hivert et al., 2018). ${\bar{H}}_{E}$ was estimated per population from the average expected heterozygosity of all SNPs within a 50,000 bp window, including invariant sites as 0s in the calculation. This approach was relatively unbiased by depth of coverage, as ${\bar{H}}_{E}$ did not correlate with average window coverage (Figure S2). ${\bar{H}}_{E}$ was calculated directly from the VCFs using a custom R script (GitHub: ja‐Reeve/CompGenoScan/R_scripts/Heterozygosity).

### 2.5 Identifying signatures of local adaptation (within species)

Genes showing signatures of differentiation across the latitudinal gradient were identified for each species using a top‐candidate approach (Yeaman et al., 2016). Initially, *F*_ST_ outliers were identified as any SNPs with scores in the top 999th quantile. Then, the number of *F*_ST_ outliers within each gene was compared to the expected number that could have arisen by chance, which was estimated from a binomial distribution with a probability of success of 0.001 (i.e., the probability of being an outlier). Any gene that had more observed *F*_ST_ outliers than the 999th quantile of this binomial distribution was considered a top candidate for local adaptation (using qbinom in R).

### 2.6 Determining orthologs: comparing patterns between species pairs

To assess patterns consistent with convergent evolution between species pairs, candidate genes were matched to orthologs in the other species. Orthologs were identified between threespines and tubesnouts using a table compiled by (in review) using OMA (v2.3.0; Altenhoff et al., 2018; Glover et al., 2019). As the two stickleback species are more closely related and share higher sequence identity, a gapped‐alignment program (GMAP; v2017‐06‐20; Wu & Watanabe, 2005) was used to identify orthologs between threespine and ninespine. For this, any alignments with a mapping quality of <80 or a percentage identity <90% were filtered out. Additionally, any genes with multiple matches (1:many & many:many orthologs) or overlapping positions within a species were removed.

To compare population divergence among species, the average *F*_ST_ score was calculated per gene. A similar approach could not be used to compare ${\bar{H}}_{E}$ because larger windows were required to obtain sufficiently precise estimates, and multiple genes could be present within a single window. Instead, the score for the whole window was applied to each gene and if a gene’s location spanned two windows then it was assigned the score of the window where most of that gene was located. This approach produces some pseudoreplication in the data as a given gene will be present in several neighboring windows, but this should have only a minor effect, causing an overestimation of the significance of any true correlation. Given that we found less correlation in these metrics than previous studies (see Discussion), this should be a conservative approach.

### 2.7 Identifying signatures of convergent evolution

The simplest approach for detecting of patterns of convergent evolution is to look for genes that are *F*_ST_ outliers in multiple species, however this approach may miss some true signals as it is very stringent (Fraser & Whiting, 2020; Storey & Tibshirani, 2003). As a more sensitive test, the Null‐W approach (Yeaman et al., 2016) was used to detect signatures of convergent evolution, by identifying top candidate genes in one species, and then comparing the *F*_ST_ scores of orthologs to the top candidate genes to a null distribution of randomly chosen genes from the genome. This was done using a standard set of 10,000 randomly chosen control SNPs and comparing both the orthologs and the null distribution genes to the control SNPs using Wilcoxon ranked sum test W‐scores (Wilcoxon, 1945; for more details see Reeve, 2019 or Supp. Mat. of Yeaman et al., 2016). These W‐scores were normalized into Z‐scores using a formula from Whitlock and Schluter (2009, p. 342), and empirical P‐values for the orthologs were calculated based on their position in the null distribution using the *empPvals* function of the *qvalue* R package (Storey et al., 2015). Empirical *p*‐values were corrected to reduce false discoveries using a Bonferroni correction. Any gene pairs that remained significant were considered signatures of convergent evolution.

## 3 RESULTS

Whole genome sequencing yielded 3.9 million threespine SNPs and 3.5 million tubesnout SNPs with consistent coverage and quality after filtering (see Table S3 for summary statistics and Figure S1 for distributions). Only 0.7 million ninespine SNPs were detected after filtering, likely as the result of the low depth of coverage for one ninespine population (NsABm).

### 3.1 Comparison of genome‐wide patterns

**FIGURE 2:**
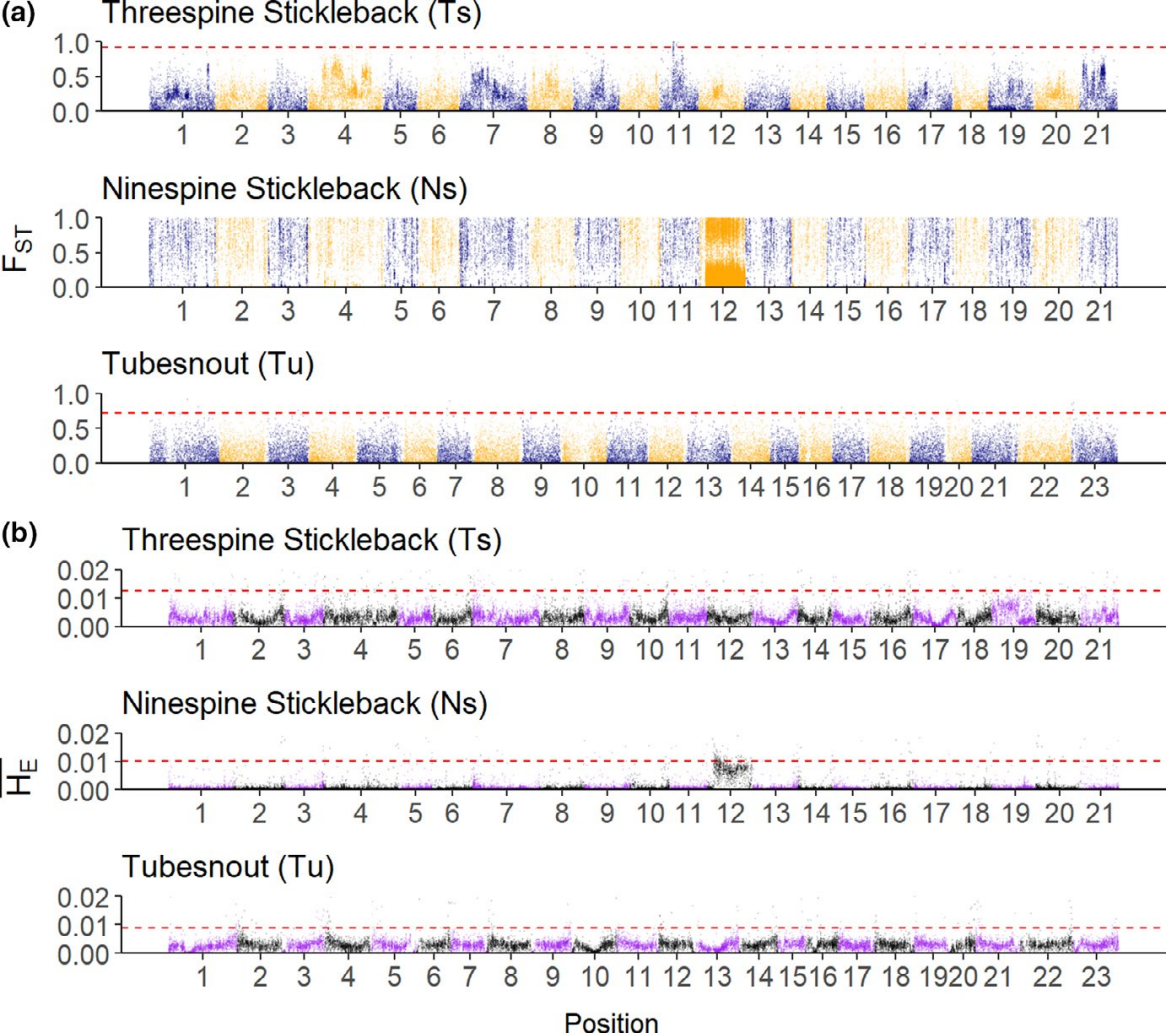
Genome wide patterns of genetic diversity within the threespine stickleback, ninespine stickleback and tubesnout. (a) *F*_ST_ per SNP and (b) ${\bar{H}}_{E}$ per 50 Kb window for each species, excluding windows in intergenic regions. Ninespine scores were mapped onto their position on the threespine genome. Threespine and tubesnout *F*_ST_ was downsized by sampling every 100th SNP along the genome, and approximately 70 windows were filtered out of the ${\bar{H}}_{E}$ plots for visual clarity. The red‐dashed lines show the 999th *F*_ST_ and 99th ${\bar{H}}_{E}$ quantiles. This plot was generated in R using the ggplot and gridExtra packages

On a genome‐wide level, average intraspecific *F*_ST_ and ${\bar{H}}_{E}$ were found to be relatively similar between the threespine and tubesnout (Table 1), but ninespine ${\bar{H}}_{E}$ was tenfold lower and *F*_ST_ was almost four times higher (Table 1). Patterns of variation in these summary statistics involved longer “genomic islands” with elevated *F*_ST_ and lower ${\bar{H}}_{E}$ in the threespine compared to the tubesnout (Figure 2; 99th *F*_ST_ quantile threespine = 0.67, tubesnout = 0.55; 99th ${\bar{H}}_{E}$ quantile threespine = 0.0070, tubesnout = 0.0056). Patterns of *F*_ST_ in ninespines were extremely heterogenous to the point that no peaks could be identified, and ${\bar{H}}_{E}$ was noticeably lower than the other two species (Figure 2) with the exception of the sex chromosome (i.e., chr12; Shapiro et al., 2009; Shikano et al., 2013).

### 3.2 Comparison of gene‐by‐gene level patterns

**FIGURE 3:**
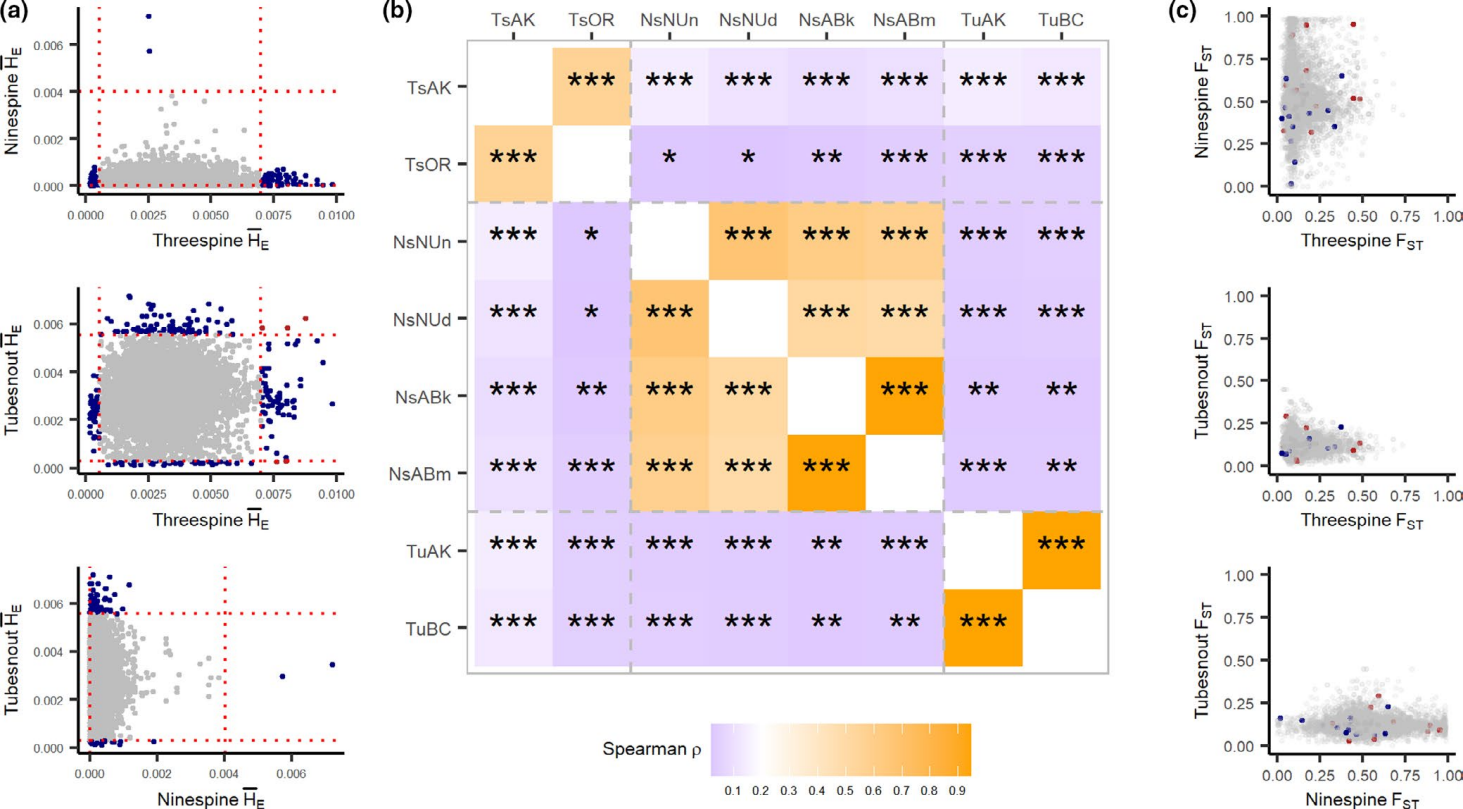
Comparison of genomic patterns among species. (a) shows the relationship in average genetic diversity (${\bar{H}}_{E}$) among genes for each species pair. Each point is a gene which is orthologous among the species. The dashed lines represent the 95th and 5th quantile of ${\bar{H}}_{E}$ in each species. Any points on the bottom left or top right segments of a panel are genes with extreme ${\bar{H}}_{E}$ that are shared among species. (b) is a matrix of ${\bar{H}}_{E}$ Spearman’s correlations among all population pairs, where the colour represents Spearman’s ρ and the text shows the significance level of a correlation test (*p < .05; **p < .01; ***p < .001). (c) shows the relationship and between the average *F*_ST_ per gene for each species. Colored points are signatures of local adaptation for each species; red for threespine sticklebacks and blue for tubesnouts. Gray points are genes not associated with local adaptation; they are partially transparant to show overlapping genes. No signatures of selection overlapped among species

At a gene‐by‐gene level, there was no clear relationship among average *F*_ST_ and ${\bar{H}}_{E}$ for orthologous genes for any species pair (Figure 3). Average *F*_ST_ per gene was weakly correlated among all species pairs, with tubesnouts and ninespines having a negative albeit non‐significant correlation (Table 2). A lack of similarity was also observed with ${\bar{H}}_{E}$ scores, with a slightly stronger negative correlation between threespines and ninespines (Table 2). Additionally, pairwise comparisons between populations showed less similarity in ${\bar{H}}_{E}$ for among‐species comparisons (*ρ* < 0.2) than within‐species comparisons (*ρ *> 0.4; Figure 3b). No clear visual pattern exists in ${\bar{H}}_{E}$ (Figure 3a) or *F*_ST_ (Figure 3c), with the exception of a flattening of ${\bar{H}}_{E}$ and elongation of *F*_ST_ towards the ninespine axes. Overall, these patterns show broad‐scale similarity between threespines and tubesnouts, which does not extend to the local gene level, or overlap with ninespines.

**TABLE 2:** Spearmen’s *ρ* correlations among species

Comparison	*F*_ST_		${\bar{H}}_{E}$		Number of genes
	*ρ*	*p*‐value	*Ρ*	*p*‐value	
Threespine vs. Ninespine	0.01	.10	−0.07	2.2 × 10^−6^	20,155
Threespine vs. Tubesnout	0.02	.04	0.09	2.2 × 10^−6^	9155
Ninespine vs. Tubesnout	−0.04	2.5 × 10^−4^	−0.02	.08	8086

Note

Correlations are made between the average *F*_ST_ and ${\bar{H}}_{E}$ of interspecific gene pairs. ${\bar{H}}_{E}$ scores are averaged across all populations before comparing species. See Figure 3b for ${\bar{H}}_{E}$ correlations among populations.

### 3.3 Testing for signatures of convergent evolution

**FIGURE 4:**
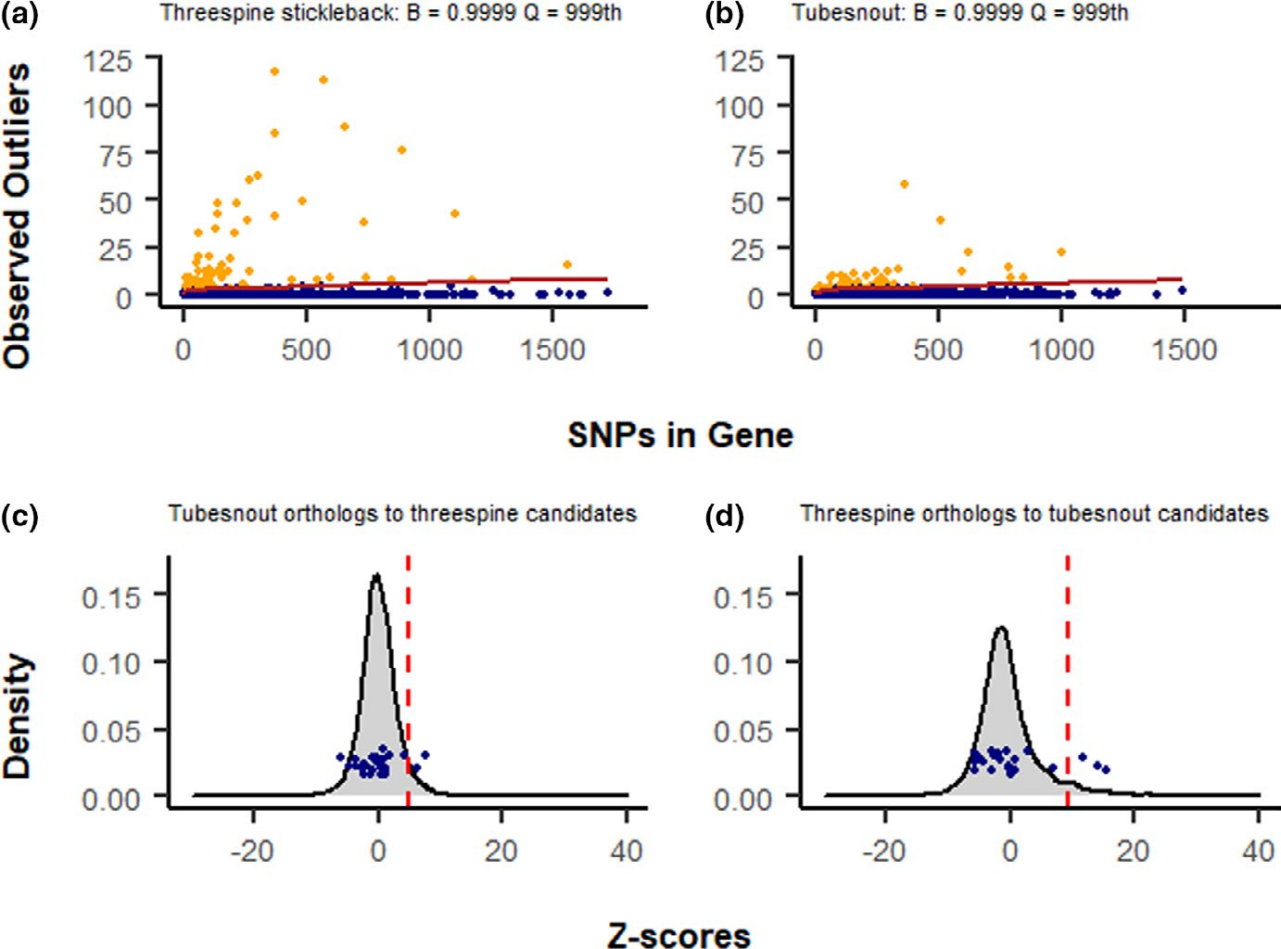
Detecting genes with elevated divergence and testing for signatures of convergent evolution. (a, b) Show the top‐candidate approach where each point is a separate gene. The total number of SNPs is compared to the number of SNP outliers in each gene, with top candidates identified as those genes that exceed the number of outliers expected under a binomial distribution, represented by the jagged red line. (c, d) Null‐W test results between (c) tubesnout orthologs of threespine top candidates and (d) threespine orthologs of tubesnout top candidates. The grey curve is the null‐distribution of Z‐scores from all orthologs of candidate genes in the focal species (i.e., tubesnout orthologs in c and threespine orthologs in d). The blue points are top‐candidate‐orthologs, whose values on the y‐axis have been jittered for visual clarity. The red dashed line is the 95th quantile of Z‐scores. FDR corrections are not shown

Northern and southern populations of each species were analyzed for genetic patterns driven by adaptation to some unmeasured factor related to latitude, by searching for genes with abnormally high patterns of *F*_ST_. Using the top candidate approach (Yeaman et al., 2016) 73 genes had extreme values of *F*_ST_ in threespines compared with 65 genes in tubesnouts (Table S4; Figure 3A). None of the top candidates were directly shared between these species, but a pair of candidate genes encoding proteins in the forkhead box family were detected (Ts: *foxo3b*; Tu: *foxb2*; Table S4). This protein family is known to influence gonad development in fish, but it is also known to have a high number of duplications (Yuan et al., 2014), so any similarities may be spurious. No signatures of high *F*_ST_ could be detected in ninespines because too many scores were close to *F*_ST_ = 1 to identify meaningful outliers. Additionally, comparing all species, only three ${\bar{H}}_{E}$ scores overlapped in the upper 95% of the distribution (Figure 3a). The Null‐W test identified five possible signatures of convergent evolution between threespines and tubesnouts (Figure 4b), but after adjusting for false discoveries these signatures lost significance (Table S5). The Null‐W test did not identify any forkhead box genes as candidates.

## 4 DISCUSSION

Comparing the results of three genome scans we found few similarities in genomic patterns among species. Only the tubesnout and threespine had similar genome‐wide average *F*_ST_ and ${\bar{H}}_{E}$ scores, but these similarities did not extend to gene‐level patterns, while comparison to the ninespine found no similarities at the genome‐wide or gene levels. Additionally, we found contrasting genome scan patterns for each species and no strong evidence to support convergent evolution. Similar absences of parallelism have been found in other comparisons of more closely related species (see below). This study highlights how the complexities of evolutionary histories, such as genetic bottlenecks or gene flow from unsampled habitats, can complicate the comparisons of genome scans.

### 4.1 Genetic patterns within each species

The patterns of genetic diversity along the threespine genome have previously been described in studies of divergence between marine and freshwater threespine population pairs (Chan et al., 2010; Hohenlohe et al., 2010; Jones et al., 2012; Roesti et al., 2014). *F*_ST_ scores typically cluster in several broad peaks in comparisons among freshwater and marine environments, with pronounced peaks around the *Eda* locus (chr4; Hohenlohe et al., 2010) and the *Pitx1* locus (chr7; Chan et al., 2010), which are involved in freshwater adaptation. Additionally, broad peaks found at three inversions (chr1, 11 and 21) have also been associated with freshwater adaptation (Jones et al., 2012; Roesti et al., 2014). Unexpectedly, as we compared two marine populations, we identified some of these characteristic patterns of marine‐freshwater divergence in this study (Figure S3). A possible explanation is that the northern and southern populations differ in the degree to which they receive gene flow from freshwater populations. In the south, threespines were sampled from an isolated stream that drained directly into the ocean, while the northern threespines were sampled from a lake connected to an estuary (Tables S1). Counterintuitively, the patterns we found probably came from freshwater alleles in the southern population, as a previous study of the lake in the north found no evidence of hybridization between “anadromous” and freshwater populations (Drevecky et al., 2013), and a study of marine populations in the North‐West Pacific found a higher frequency of freshwater associated alleles at the *EDA* locus in Oregon than Alaska (Morris et al., 2018). However, to test such hypotheses about introgression, we would have to look at the frequency of the low‐plate *EDA* allele and the frequencies of inversions in Oregon and Alaska and contrast this with nearby freshwater populations. An alternative explanation is that the some of the patterns of marine‐freshwater adaptation may also be pleiotropically connected to thermal regulation, as has been suggested for the *EDA* locus (Morris et al., 2018). Whether it is differential gene‐flow or pleiotropic adaption, we have found that the genomic landscape of geographically diverse marine threespines is strikingly similar to the marine‐freshwater landscape.

In contrast to the patterns found in threespines, no large peaks of *F*_ST_ were present along the tubesnout genome (Figure 2). Instead, there were several small and narrow *F*_ST_ peaks suggesting that the tubesnout genome has been shaped by processes that do not leave strong genetic signals, such as genetic drift or polygenic adaptation (Rockman, 2012; Stinchcombe & Hoekstra, 2008; Yeaman, 2015). As the Null‐W test is designed to detect linked clusters of *F*_ST_ outliers, this also explains the lack of any signatures of convergent evolution. Since the patterns of *F*_ST_ were not strongly heterogeneous in tubesnout, it is unsurprising that no significant matches to threespine were found.

The genetic patterns present in the ninespine stickleback were likely the result of a strong genetic bottleneck and isolation between the northern and southern populations, as on average, genetic divergence was high and genetic diversity was low in all four populations (Table 1, Figure 2). Southern populations were sampled from two prairie lakes, which were formed when a larger post‐glacial lake dried up, isolating these ninespine populations and presumably causing a genetic bottleneck (Tufts, 2018), similar to the founder‐effect observed in Nordic populations (Shikano et al., 2010). In contrast, the northern populations were sampled from lakes close to the sea, which potentially has provided several opportunities for gene flow from the marine populations. A phylogeographic study separated ninespine populations from the Atlantic coast and Great Lakes regions into two post‐glacial lineages, with evidence suggesting that the divergence time among these lineages may be much older than the last glacial maximum (Aldenhoven et al., 2010). Presumably, the prairie lake populations are part of this Great Lakes lineage (Tufts, 2018) and therefore should be highly diverged from the Northern populations. The extreme genetic divergence among these populations is likely to be the result of long‐term genetic isolation combined with a strong genetic bottleneck in the southern populations, not adaptation to latitude.

Comparing the genome scans of all species reveals three distinct patterns, suggesting that the balance between the evolutionary processes has differed among these species. The *F*_ST_ Manhattan plots (Figure 2a) show different patterns, which can be interpreted as the result of three distinct evolutionary scenarios: local adaptation (threespine), genetic bottlenecks (ninespine) and a weak or polygenic selection and/or drift (tubesnout). This does not imply that the ninespine has not experienced selection or that the threespine has not been affected by drift, just that the patterns of diversity in the genome have been more strongly affected by different processes in each species.

A major caveat to these results is that very few populations were sampled per species. Pool‐seq mixes alleles across a population, which means that the basic sampling unit is a population, in effect each species had only two to four data points. The comparisons made in this study may have been underpowered to detect any shared genetic patterns. However, the presence of threespine peaks in previously identified regions undergoing adaptation (Figure S3) shows that strong genetic patterns were detectable, thus only subtle patterns of genetic diversity may have been lost. The lack of this pattern in tubesnout may be due to the lack of an evolutionary history of repeated colonization followed by gene‐flow from freshwater populations, which can lead to complex genomic architecture for adaptive traits (Faria et al., 2019; Tigano & Friesen, 2016). All things considered; this study demonstrates the diversity of genetic patterns that can be identified from genome scans of wild species, even with a limited number of populations.

### 4.2 Comparative genome scans in a broader context

In many cases, similarity in patterns revealed by genome scans among species decreases with phylogenetic distance. Divergent populations of the same species, and sister species that have recently diverged, often have more strongly shared genetic patterns (Burri et al., 2015; Fischer et al., 2013; Ravinet et al., 2016; Renaut et al., 2013; Vijay et al., 2016; Westram et al., 2014). At greater phylogenetic distances, species that diverged long ago often show less similarity in their genetic patterns, with most of the residual patterns being attributed to convergent evolution (Raeymaekers et al., 2017; Vijay et al., 2017; Le Moan et al., 2019, bioRxiv). Henderson and Brelsford (2020) studied this contrast explicitly in three hummingbird species‐pairs, showing that more distantly related species pairs had reduced correlations in genetic diversity and increased *F*_ST_ across the genome. Similarly, a meta‐analysis (Conte et al., 2012) demonstrated a negative relationship between the proportion of shared signatures of trait variation and the time since divergence of both species and population pairs. Shared patterns of genome scan variation is not a universal outcome, as Raeymaekers et al. (2017) showed no shared genetic patterns among species despite significant phenotypic sharing. Our study fits in with this latter category, without any signatures of convergent evolution and widespread differences in genetic patterns along the genome.

In addition to any effect related to phylogenetic distance, local adaptation to marine or freshwater environments might have also contributed to the greater genome‐wide correlations in *F*_ST_ and ${\bar{H}}_{E}$ between threespines and ninspines, relative to the tubesnout. Although we sampled threespines from marine populations, they harbor freshwater adapted alleles (Schluter & Conte, 2009), and a few similar genes may also underpin freshwater adaptation in some ninespine populations (Wang et al., 2020). Thus, the greater similarity in genome‐wide patterns may also be related to broad similarity in selection pressures across the marine‐freshwater gradient, even though we sampled marine threespines and freshwater ninespines. In contrast, tubesnout would not share these genetic patterns as they are an exclusively marine species.

An interesting contrast to the results of this study is Vijay’s et al. (2017) study of the long‐term conservation of genomic patterns among three species of birds. They compared species that had similar generation and divergence times to the fishes used in this study (Bird clades in Vijay et al. = 23–55 mya; threespine to ninespine = 26 mya [Varadharajan et al., 2019]; threespine to tubesnout = 50 mya [Betancur et al., 2013]); suggesting that patterns of genetic diversity are conserved long past speciation. Vijay found stronger correlations in genetic diversity among their species pairs (range of Pearson’s *r* = 0.08–0.27) than were found in this study (range Spearman’s *ρ* = −0.07–0.09). However, Manhattan plots of *F*_ST_ and genetic diversity also did not show any clear overlapping peaks or troughs (Figure 2). Other studies looking at fewer genetic markers have also identified more conserved levels of genetic diversity in birds than fishes (Adams & Hadly, 2013; Johns & Avise, 1998), possibly as the result of a faster genome‐averaged mutation rate, which has been observed between teleosts and mammals (Ravi & Venkatesh, 2008). Alternatively, fish genomes may evolve faster than birds due to differences in their recombination map or gene densities. Investigating the differences in the rates of evolution among broad taxonomic groups is an interesting question, which is now possible with the increase in publicly available whole genome data.

## 5 CONCLUSION

In some ways the lack of shared genetic patterns among species is not surprising, as evolution is a balance of several forces that leave a complex mosaic of patterns in the genome. Finding any common patterns among species would require very strong evolutionary forces to consistently shift this balance in the same way for every species. When comparing genome scans divergence in such patterns may be the norm and convergence may be a comparatively rare exception. Our results demonstrate that genome scans can be noisy, due to the effects of demographic shifts, genomic architecture or selective sweeps. Yet these noisy results help in the development of a general theory on how evolutionary forces shape the genome, by showing when similarities do not arise and some of the oddities that one may see when performing a genome scan.

## CONFLICT OF INTEREST

None of the authors have conflict of interest.

## AUTHOR CONTRIBUTIONS

**James Reeve:** Conceptualization (equal); Data curation (lead); Formal analysis (lead); Investigation (equal); Project administration (lead); Resources (equal); Visualization (lead); Writing – original draft (lead); Writing – review & editing (equal). **Qiushi Li:** Methodology (supporting); Resources (equal); Supervision (supporting); Writing – review & editing (equal). **Dorothea Lindtke:** Resources (equal); Supervision (supporting); Writing – review & editing (supporting). **Samuel Yeaman:** Conceptualization (equal); Funding acquisition (lead); Investigation (equal); Project administration (supporting); Supervision (lead); Writing – review & editing (equal).

## Supporting information

Supplementary Material

## Data Availability

The raw sequencing data for each population has been uploaded to NCBI’s sequence read archive (NCBI SRA BioProject: PRJNA776244) and all code used in the analysis is uploaded to GitHub: ja‐Reeve/CompGenoScan.
