# Supplementary material for: Comparing genome scans among species of the stickleback order reveals three different patterns of genetic diversity
Source: Ecol Evol. 2022 Jan 24;12(1):e8502. doi: 10.1002/ece3.8502 (PMC8796908; doi:10.1002/ece3.8502)
Supplement: Supplementary file 1 — Supplementary Material [file ECE3-12-e8502-s001.docx]

**Table S1:** Description of sampling sites, including the number of samples collected at each site. For sites that were sampled by independent collectors, the coordinates are estimated as the centre of the lake or a nearby beach for marine sites. The descriptions are brief summaries of the habitat the samples were collected in. The ninespine samples were collected through a mixture of seine and dip netting and minnow trapping (Tufts, 2018). N represents the number of samples collected at each location.

| Pop ID | Latitude  Longitude | Site Name | Species | N | Method | Description |
| --- | --- | --- | --- | --- | --- | --- |
| TsAK | 61.5975  -149.3458 | Mud Lake | *Gasterosteus aculeatus* | 52 | Minnow trap | Freshwater lake connected to Cook Inlet, Alaska^1^ |
| TsOR | 43.3432  -124.3499 | Bastendorff Beach | *Gasterosteus aculeatus* | 51 | Minnow trap | Creek on beach, intermittent daily contact with ocean |
| NsNUn | 66.4424  -70.7895 | Nettling Lake | *Pungitius pungitius* | 50* | All | Overflow ponds adjacent to freshwater lake |
| NsNUd | 63.7471  -68.4887 | Dead Dog Lake | *Pungitius pungitius* | 50* | All | Freshwater lake outskirts of Iqaluit, Nunavut |
| NsABk | 54.0496  -110.9017 | Kehiwin Lake | *Pungitius pungitius* | 30 | All | Freshwater prairie lake |
| NsABm | 54.2415  -110.9109 | Moose Lake | *Pungitius pungitius* | 50* | All | Freshwater prairie lake |
| TuAK | 58.3808  -134.6917 | Auke Nu Cove | *Aulorhynchus flavidus* | 50 | Seine net | Bay north of Juneau, Alaska |
| TuBC | 48.6543  -123.3931 | Port Sidney Marina | *Aulorhynchus flavidus* | 50 | Dip net | End of pier inside marina |

*Sample number (N) is the number of samples donated for sequencing. The original collection numbers from these lakes are found in Tufts (2018).

^1^Karve, von Hippel and Bell, 2008

**Table S2:** Summary of pools used for sequencing. All quality and quantity measurements are the average of three measurements. A260/A280 and A260/A230 are the Nanodrop quality scores which indicate potential protein or organic salt contamination. Explanations of the Pop IDs are in Fig. 1 of the main text.

| **Pop ID** | **Pool size (N)** | **Ploidy (2N)** | **Mass per individual (ng)** | **Concentration of Pool (ng/μL)** | **Volume of Pool (μL)** | **A260/**  **A280** | **A260/**  **A230** |
| --- | --- | --- | --- | --- | --- | --- | --- |
| *Threespine stickleback* | | | | | | | |
| TsAK | 51 | 102 | 109.8 | 36.3 | 171.5 | 1.913 | 2.262 |
| TsOR | 52 | 104 | 107.7 | 38.2 | 172.5 | 1.878 | 2.558 |
| *Ninespine stickleback* | | | | | | | |
| NsNUn | 46 | 92 | 65.2 | 16.6 | 177.8 | 1.854 | 1.532* |
| NsNUd | 42 | 84 | 71.4 | 19.1 | 161.0 | 1.916 | 2.869 |
| NsABk | 30 | 60 | 73.3 | 14.2 | 170.7 | 1.830 | 2.241 |
| NsABm | 41 | 82 | 51.2 | 7.8 | 167.2 | 1.833 | 1.999 |
| *Tubesnout* | | | | | | | |
| TuAK | 44 | 88 | 59.1 | 13.1 | 192.3 | 1.969 | 2.229 |
| TuBC | 50 | 100 | 52.0 | 14.4 | 197.0 | 1.946 | 2.292 |

*This pool failed the minimum quality threshold

**Table S3:** Summary statistics for filtered SNP quality and coverage. The central tendency of each population is represented by the median, with variance given as the median average difference (MAD) scores. Variables were extracted from the VCF files and analysed in R. **GQ** = genotype (SNP) quality; **DP** = depth of coverage. Pool size is the number of fish in each population.

| Population | Pool Size | Number of SNPs | Median GQ | MAD (GQ) | Median DP | MAD (DP) |
| --- | --- | --- | --- | --- | --- | --- |
| *Threespine stickleback* | | | | | | |
| TsAK | 52 | 3,928,772 | 127 | 77.10 | 82 | 11.86 |
| TsOR | 51 |  | 115 | 71.16 | 86 | 11.86 |
| *Ninespine stickleback* | | | | | | |
| NsNUn | 46 | 687,627 | 155 | 106.75 | 111 | 19.27 |
| NsNUd | 42 |  | 134 | 75.61 | 90 | 16.31 |
| NsABk | 30 |  | 142 | 123.06 | 75 | 16.31 |
| NsABm | 41 |  | 109 | 81.54 | 58 | 8.90 |
| *Tubesnout* | | | | | | |
| TuAK | 44 | 3,466,658 | 107 | 66.72 | 79 | 11.86 |
| TuBC | 50 |  | 120 | 69.68 | 92 | 13.34 |

**Table S4 (Next page):** Gene annotations for signatures of local adaptation for the threespine stickleback (left) and tubesnout (right). Gene IDs are based on the Broad stickleback genome annotations v96, and the gene names were searched on the stickleback genome browser: <https://stickleback.genetics.uga.edu/> (accessed 2020/11/12). Tubesnout annotations were based on threespine genome orthologs that were detected by Li et al. (in review). Any tubesnout genes with ENSEMBL IDs labelled as “none” did not have any threespine orthologs identified.

| **Threespine stickleback** | | | | | **Tubesnout** | | | | | |
| --- | --- | --- | --- | --- | --- | --- | --- | --- | --- | --- |
| **Chr** | **Start position** | **End position** | **Broad ID** | **Gene** | **Chr** | **Start position** | **End position** | **ID** | **Threespine Broad ID** | **Gene** |
| I | 11805780 | 11855863 | ENSGACG00000010697 | ncam1b | *1* | 13777255 | 13857990 | jg10055.t1 | none | - |
| III | 8930906 | 8941593 | ENSGACG00000012221 | - |  | 16644721 | 16811595 | jg10239.t1 | none | - |
| IV | 7180013 | 7181304 | - | - |  | 18354901 | 18368943 | jg10291.t1 | none | - |
|  | 8327962 | 8332475 | ENSGACG00000009819 | Chico |  | 18539842 | 18558540 | jg10306.t1 | none | - |
|  | 8338620 | 8357409 | ENSGACG00000009827 | gna11b |  | 21817979 | 21895613 | jg10502.t1 | none | - |
|  | 8375252 | 8378520 | ENSGACG00000009899 | pdcd10b |  | 22216667 | 22256832 | jg10517.t1 | none | - |
|  | 11851618 | 11864625 | ENSGACG00000011719 | TATDN3 |  | 24966746 | 24980382 | jg10675.t1 | none | - |
|  | 11851618 | 11864625 | ENSGACG00000011714 | si:dkey-36g24.3 |  | 424767 | 451123 | jg1571.t1 | none | - |
|  | 11864859 | 11868857 | ENSGACG00000011736 | fuca2 | *2* | 1381503 | 1391092 | jg8883.t1 | ENSGACG00000001431 | - |
|  | 11877099 | 11883636 | ENSGACG00000011743 | hivep2b |  | 1360896 | 1371836 | jg8881.t1 | none | - |
|  | 14741484 | 14743751 | - | - |  | 16447870 | 16484330 | jg9653.t1 | none | - |
| V | 6506443 | 6516784 | ENSGACG00000002758 | - | *3* | 9053345 | 9089728 | jg11786.t1 | ENSGACG00000020028 | - |
| VI | 9349293 | 9386955 | ENSGACG00000000924 | srgap3 | *4* | 7260281 | 7286250 | jg713.t1 | ENSGACG00000007781 | coq6 |
|  | 9407744 | 9424634 | ENSGACG00000000920 | rad18 | *5* | 4163743 | 4165719 | jg29177.t1 | none | - |
| VII | 8529496 | 8560088 | ENSGACG00000008910 | foxo3b |  | 5078292 | 5088070 | jg29111.t1 | none | - |
| VIII | 2588538 | 2590641 | ENSGACG00000013653 | pnrc2 |  | 5095533 | 5112401 | jg29109.t1 | none | - |
|  | 5593737 | 5597358 | - | - | *6* | 2295090 | 2389053 | jg18436.t1 | ENSGACG00000006690 | TNFRSF11B |
|  | 10804454 | 10808504 | - | - |  | 5466068 | 5509023 | jg18265.t1 | ENSGACG00000005006 | usp45 |
|  | 13026137 | 13089933 | ENSGACG00000006485 | dnah5 | *8* | 922368 | 991958 | jg3812.t1 | ENSGACG00000018525 | - |
|  | 13716709 | 13720863 | - | - |  | 4838031 | 4847101 | jg3927.t1 | ENSGACG00000018729 | fam199x |
| IX | 10643230 | 10653863 | ENSGACG00000002714 | crispld1a | *10* | 293375 | 297973 | jg13874.t1 | ENSGACG00000007973 | foxb2 |
| X | 14277363 | 14325662 | ENSGACG00000016239 | smyd3 |  | 4645128 | 4655617 | jg13500.t1 | ENSGACG00000003804 | top3b |
|  | 14332970 | 14394705 | ENSGACG00000016241 | kif26ba |  | 5332377 | 5350105 | jg13447.t1 | ENSGACG00000003366 | arrdc1a |
| XI | 6183917 | 6199455 | ENSGACG00000014911 | ca8 |  | 4345063 | 4382648 | jg13526.t1 | ENSGACG00000003927 | dhx37 |
|  | 6240142 | 6258564 | - | - |  | 16294115 | 16298849 | jg12960.t1 | none | - |
|  | 6261066 | 6269665 | - | - | *11* | 12188831 | 12217966 | jg8304.t1 | ENSGACG00000019059 | - |
|  | 6269749 | 6273261 | - | - |  | 10335645 | 10349330 | jg8179.t1 | ENSGACG00000018436 | - |
|  | 6276992 | 6284246 | - | - |  | 10353694 | 10364028 | jg8180.t1 | ENSGACG00000018434 | rtn4rl2a |
|  | 6284575 | 6287622 | ENSGACG00000014919 | cryz |  | 9620041 | 9626019 | jg8136.t1 | ENSGACG00000018198 | ENDOV |
|  | 6288915 | 6296923 | ENSGACG00000014921 | dars2 |  | 11288265 | 11373845 | jg8242.t1 | none | - |
|  | 6296957 | 6302132 | ENSGACG00000014929 | impad1 | *12* | 2688271 | 2711131 | jg22872.t1 | ENSGACG00000011760 | GHRHR |
|  | 6302306 | 6305411 | ENSGACG00000014942 | ankrd45 |  | 20390752 | 20426965 | jg23904.t1 | none | - |
|  | 6306759 | 6311297 | ENSGACG00000014944 | trnau1apb |  | 10555724 | 10571654 | jg23368.t1 | none | - |
|  | 6314448 | 6316797 | ENSGACG00000014947 | fitm1 | *13* | 6540809 | 6550322 | jg27042.t1 | ENSGACG00000017665 | kcnj9 |
|  | 6327476 | 6338771 | ENSGACG00000014948 | myh7l |  | 4065025 | 4079700 | jg26850.t1 | none | - |
|  | 6344052 | 6357478 | ENSGACG00000014960 | myh7 |  | 1117210 | 1121263 | jg26660.t1 | none | - |
|  | 6365042 | 6378736 | - | - |  | 5129406 | 5133624 | jg26914.t1 | none | - |
|  | 6383759 | 6386994 | - | - |  | 5139102 | 5152252 | jg26915.t1 | none | - |
|  | 6408156 | 6416746 | - | - |  | 6073876 | 6080298 | jg26999.t1 | none | - |
|  | 6400129 | 6401355 | - | - |  | 1078448 | 1098177 | jg26654.t1 | none | - |
|  | 6449217 | 6453975 | ENSGACG00000014969 | - | *14* | 9189050 | 9214971 | jg15807.t1 | ENSGACG00000011899 | ndufb10 |
|  | 6459135 | 6472605 | ENSGACG00000014972 | klhl20 |  | 8008000 | 8028717 | jg15746.t1 | ENSGACG00000012403 | adap2 |
|  | 6503679 | 6509033 | - | - |  | 7956238 | 7967129 | jg15741.t1 | none | - |
|  | 6511106 | 6513248 | - | - |  | 8164916 | 8170003 | jg15755.t1 | none | - |
|  | 6518783 | 6519332 | - | - |  | 11671450 | 11683782 | jg15974.t1 | none | - |
|  | 6578279 | 6608906 | ENSGACG00000014996 | - | *15* | 6257973 | 6301021 | jg3242.t1 | none | - |
|  | 6578279 | 6608906 | ENSGACG00000014993 | - |  | 7906560 | 7920669 | jg3095.t1 | ENSGACG00000016803 | elp1 |
|  | 7738181 | 7742146 | - | - |  | 12217940 | 12244718 | jg2871.t1 | none | - |
|  | 7792972 | 7858948 | ENSGACG00000015386 | epha4b | *16* | 916560 | 921189 | jg2327.t1 | none | - |
|  | 8510520 | 8514173 | - | - |  | 5271907 | 5311085 | jg7127.t1 | none | - |
|  | 8531099 | 8567751 | ENSGACG00000015576 | kcnn1a |  | 5206656 | 5264544 | jg7128.t1 | none | - |
| XII | 7972945 | 8046739 | ENSGACG00000017532 | atp8a1 | *17* | 9369929 | 9380427 | jg22166.t1 | none | - |
| XIV | 11780134 | 11789992 | ENSGACG00000006739 | fra10ac1 | *18* | 18919677 | 18933321 | jg16421.t1 | ENSGACG00000006482 | swsap1 |
| XVI | 4753578 | 4764120 | ENSGACG00000019360 | - |  | 8631084 | 8645933 | jg17183.t1 | ENSGACG00000003575 | gucy2g |
|  | 4753578 | 4764120 | ENSGACG00000019358 | si:dkey-38p12.3 |  | 1954586 | 1965099 | jg17522.t1 | none | - |
|  | 4780300 | 4792453 | ENSGACG00000019362 | neurl4 |  | 7148250 | 7152291 | jg17270.t1 | none | - |
|  | 4793754 | 4812953 | ENSGACG00000019365 | shbg | *19* | 11074030 | 11077728 | jg25922.t1 | ENSGACG00000019461 | OSBPL8 |
|  | 4871821 | 4874179 | ENSGACG00000019392 | - |  | 6280000 | 6291937 | jg26198.t1 | none | - |
| XVII | 7767504 | 7830253 | ENSGACG00000006238 | ncbp2 |  | 13307885 | 13351596 | jg25816.t1 | none | - |
|  | 7767504 | 7830253 | ENSGACG00000006258 | - |  | 6004272 | 6008738 | jg26213.t1 | none | - |
|  | 7901509 | 7950094 | ENSGACG00000006451 | - |  | 18678920 | 18736997 | jg25555.t1 | none | - |
|  | 7901509 | 7950094 | ENSGACG00000006440 | henmt1 | *20* | 7080925 | 7112826 | jg25143.t1 | none | - |
|  | 7901509 | 7950094 | ENSGACG00000006445 | - | *21* | 5819162 | 5854309 | jg18851.t1 | none | - |
|  | 19711616 | 19712362 | - | - |  | 8872704 | 8879751 | jg19081.t1 | none | - |
| XVIII | 6278048 | 6285831 | - | - |  | 13804425 | 13804826 | jg19370.t1 | none | - |
|  | 6326992 | 6330305 | ENSGACG00000003159 | eomesa (rev. std) |  | | | | | |
|  | 6326992 | 6330305 | ENSGACG00000003160 | eomesa |  |  |  |  |  |  |
|  | 6334616 | 6335352 | - | - |  |  |  |  |  |  |
|  | 6337166 | 6370293 | ENSGACG00000003175 | - |  |  |  |  |  |  |
| XIX | 13351637 | 13362700 | ENSGACG00000012911 | pold1 |  |  |  |  |  |  |
| XX | 6588281 | 6738537 | ENSGACG00000007345 | grid2 |  |  |  |  |  |  |
| XXI | 8491544 | 8500737 | ENSGACG00000017528 | si:ch73-62l21.1 |  |  |  |  |  |  |
|  | 8512047 | 8515896 | ENSGACG00000017534 | - |  |  |  |  |  |  |

**Table S5:** Null-W test results and gene annotations of signatures of convergent evolution. Threespine gene positions and IDs are taken from the Peichel et al. (2017) genome map and Broad IDs were taken from the Broad S1 gene annotations (v96). Tubesnout gene positions and IDs are taken from the tubesnout genome (v2.2) and annotations are taken from the threespine stickleback genome. Putative functions of each candidate were identified by a manual search of NCBI’s gene database.

| **Gene ID** | **Ortholog position** | **Z** | **Emp. p-value** | **Adj. p-value** | **Broad S1 ID** | **Putative function** |
| --- | --- | --- | --- | --- | --- | --- |
| **Ts -> Tu** | *Positions on the tubesnout genome and annotations on the threespine genome* | | | | | |
| *Tu_jg265.t1* | chr4:17635033 – 17654463 | 7.59 | 0.01 | 0.32 | ENSGACG00000011743 | Zinc finger. Ion transport through cell membrane* |
| *Tu_jg28127.t1* | chr22:6875393 – 6900665 | 6.34 | 0.03 | 0.62 | ENSGACT00000015565 |  |
| **Tu -> Ts** | *Positions and annotations on the threespine genome* | | | | | |
| *Ts_jg18432.t1* | chr7:10170669 - 10201471 | 14.21 | 0.02 | 0.43 | ENSGACT00000026509 | Uncharacterised gene |
| *Ts_jg25097.t1* | chr20:11865524 - 11880192 | 15.31 | 0.02 | 0.36 | ENSGACT00000008878 | TNF receptor. Related to apoptosis and inflammation |
| *Ts_jg6914.t1* | chr9:11820737 - 11836662 | 11.84 | 0.03 | 0.65 | ENSGACT00000024103 | Endonuclease V |

*Both Broad IDs match the same gene, likely representing homologues.

**Supplementary Figures:**


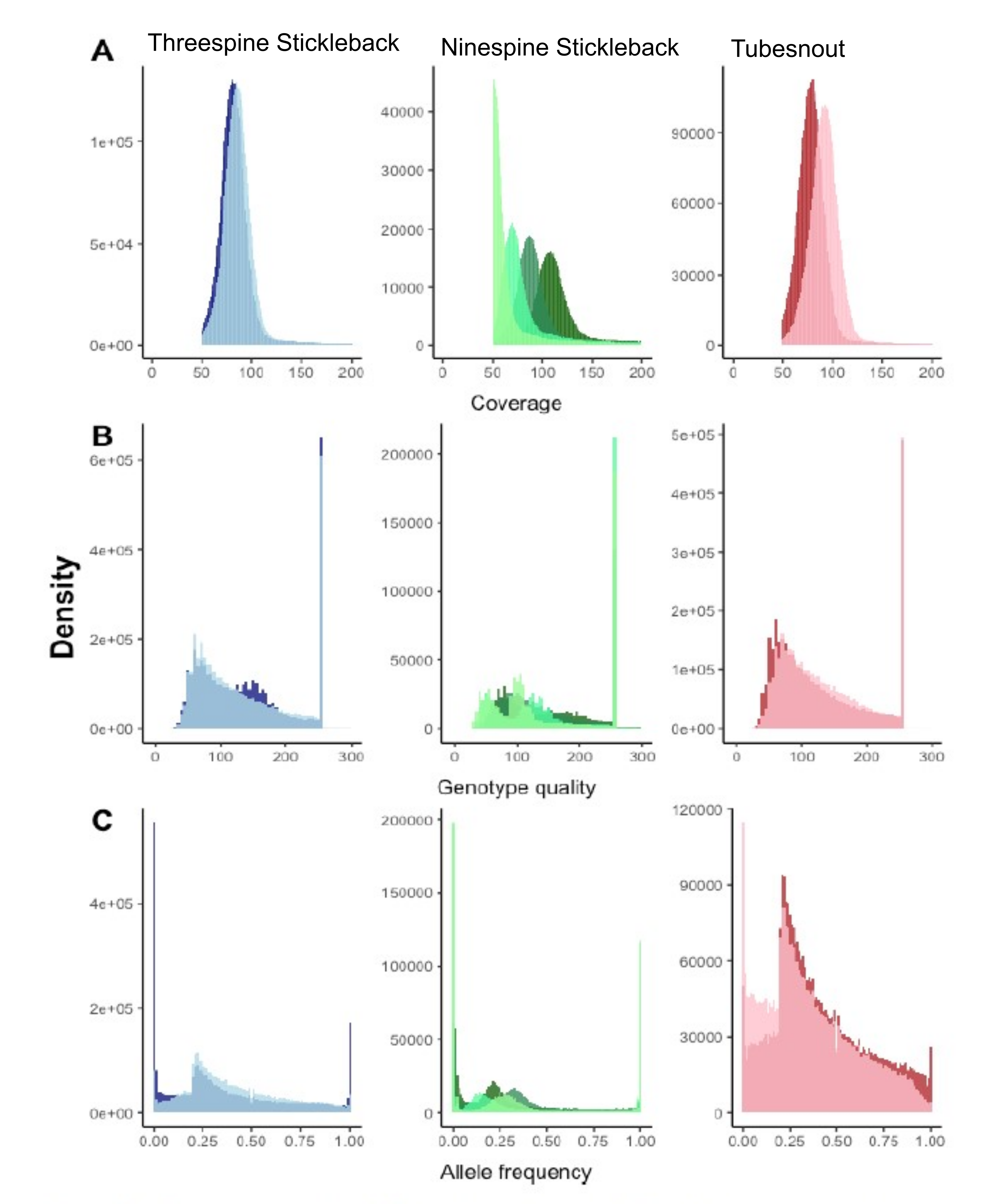


**Figure S1:** Distributions of **A)** coverage, **B)** genotype quality and **C)** allele frequencies for filtered SNPs (**Filters:** cov < 50; qual < 20; maf < 0.05; min read count < 2) in each species. Lighter shades represent southern populations.


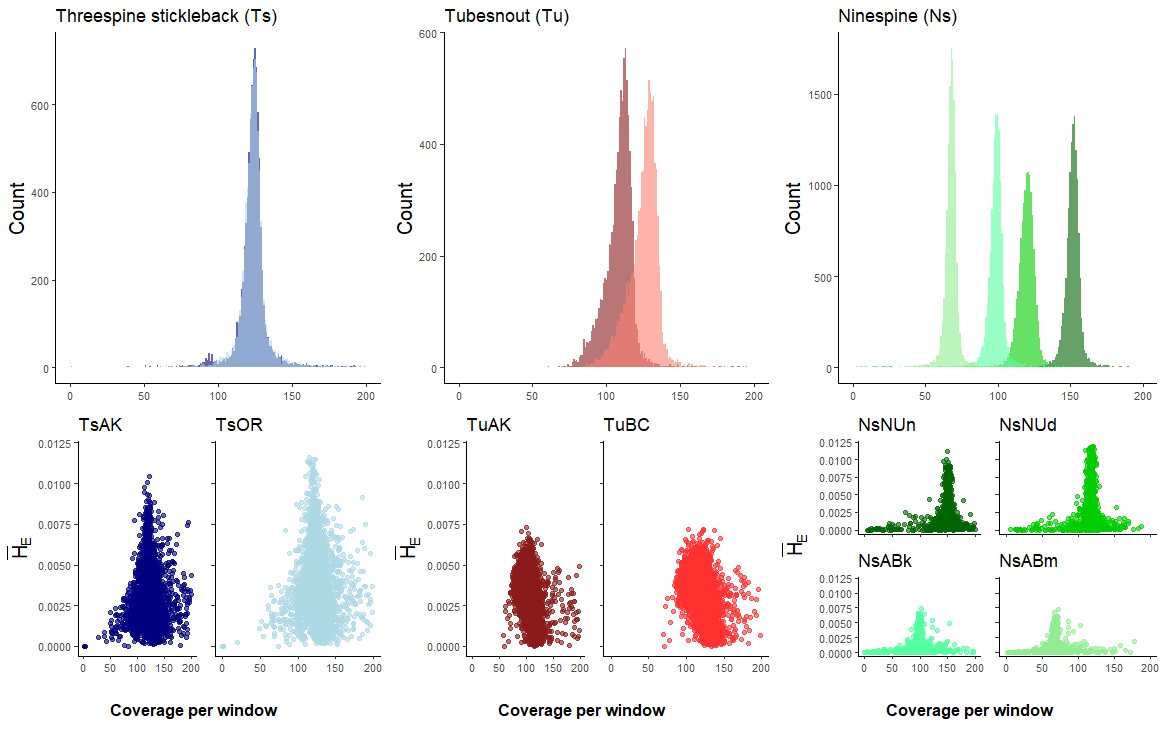
**Figure S2:** Average coverage per 50,000 bp window. The top panels are histograms of coverage with lighter colours representing the southern populations. The bottom panels are scatterplots of the average coverage vs $\bar{H}_{E}$ of each window, for each population. X-axes have been limited to cov ≤ 200 to remove a long right tail from the distribution.


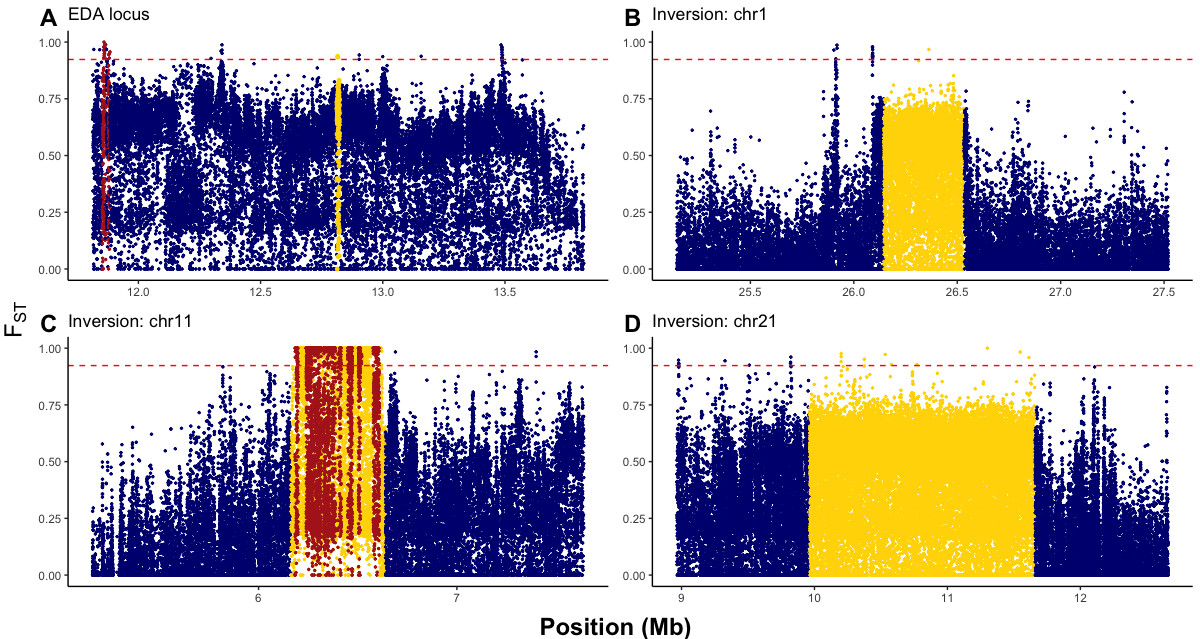


**Figure S3:** Manhattan plots investigating regions of the threespine stickleback genome known to show elevated genetic divergence among freshwater and marine ecotypes. The panels show a) the EDA locus (chr4: 12,812,614 - 12,825,836) and inversions on b) chr1, c) chr11, and d) chr21. Each focal region is highlighted in yellow, while SNPs in candidate genes from this study are highlighted in red. The positions of inversions on the Peichel genome (Peichel *et al.*, 2017) are approximated from their published positions of the Broad S1 genome (Jones *et al.*, 2012; Glazer *et al.*, 2015).

**References:**

Glazer, A. M., Killingbeck, E. E., Mitros, T., Rokhsar, D S. & Miller. C. T. (2015). Genome assembly improvement and mapping convergently evolved skeletal traits in sticklebacks with genotyping-by-sequencing. *G3*, 5, 1463–72. doi: 10.1534/g3.115.017905.

Jones, F. C., Grabherr, M. G., Chan, Y. F., Russell, P., Mauceli, E., Johnson, J., Swofford, R., Pirun, M., Zody, M. C., White, S., Birney, E., Searle, S., Schmutz, J., Grimwood, J., Dickson, M. C., Myers, R. M., Miller, C. T., Summers, B. R., Knect, A. K., … & Kingsley, D. M. (2012). The genomic basis of adaptive evolution in threespine sticklebacks. *Nature*, 484(7392), 55–61. doi: 10.1038/nature10944.

Karve, A. D., von Hippel, F. A. & Bell, M. A. (2008). Isolation between sympatric anadromous and resident threespine stickleback species in Mud Lake, Alaska. *Environmental Biology of Fishes*, 81, 287–296. doi: 10.1007/s10641-007-9200-2.

Li, Q., Lindtke, D. & Yeaman, S. (in review). Co-evolution of local adaptation and genome architecture in threespine stickleback.

Peichel, C. L., Sullivan, S. T., Liachko, I. & White, M. A. (2017). Improvement of the threespine stickleback genome using a Hi-C-based proximity-guided assembly. *Journal of Heredity*, 108(6), 693–700. doi: 10.1093/jhered/esx058.

Tufts, T. (2018). *Assessing temperature tolerance in ninespine Stickleback (Pungitus pungitus) in response to climate change*. University of Calgary.
